# Identification of miRNA-Small Molecule Associations by Continuous Feature Representation Using Auto-Encoders

**DOI:** 10.3390/pharmaceutics14010003

**Published:** 2021-12-21

**Authors:** Ibrahim Abdelbaky, Hilal Tayara, Kil To Chong

**Affiliations:** 1 Artificial Intelligence Department, Faculty of Computers and Artificial Intelligence, Benha University, Banha 13518, Egypt; ibrahim.abdelbaky@fci.bu.edu.eg; 2 School of International Engineering and Science, Jeonbuk National University, Jeonju 54896, Korea; 3 Department of Electronics and Information Engineering, Jeonbuk National University, Jeonju 54896, Korea; 4 Advanced Electronics and Information Research Center, Jeonbuk National University, Jeonju 54896, Korea

**Keywords:** miRNA-small molecule associations, drug repurposing, deep learning auto-encoders, sequence encoding

## Abstract

MicroRNAs (miRNAs) are short non-coding RNAs that play important roles in the body and affect various diseases, including cancers. Controlling miRNAs with small molecules is studied herein to provide new drug repurposing perspectives for miRNA-related diseases. Experimental methods are time- and effort-consuming, so computational techniques have been applied, relying mostly on biological feature similarities and a network-based scheme to infer new miRNA–small molecule associations. Collecting such features is time-consuming and may be impractical. Here we suggest an alternative method of similarity calculation, representing miRNAs and small molecules through continuous feature representation. This representation is learned by the proposed deep learning auto-encoder architecture. Our suggested representation was compared to previous works and achieved comparable results using 5-fold cross validation (92% identified within top 25% predictions), and better predictions for most of the case studies (avg. of 31% vs. 25% identified within the top 25% of predictions). The results proved the effectiveness of our proposed method to replace previous time- and effort-consuming methods.

## 1. Introduction

After the non-coding regions of the human genome were found to play a significant role in cells, they gained some interest in biomedical and drug research, which had until then mainly focused on protein-coding regions. Non-coding RNAs (ncRNAs) [1] can modulate gene expression levels and are linked to different biological activities and diseases in humans [2]. MicroRNAs (miRNAs) are single-stranded short non-coding RNA sequences that are 18–24 nucleotides in length. They are of special importance because of their effects on gene activity and expression at the post-transcriptional level [3]. In humans, they have been found to affect more than one third of genes [4]. A single miRNA can regulate many genes simultaneously [5,6]. Many examples of miRNAs that affect the regulation of multiple genes are found in the miRTarBase [7] and TransmiR [8] databases, such as miR-186 [9,10] and miR-148a [11,12,13,14].

The first miRNA was discovered in 1993 [15]; thereafter, numerous miRNAs have been discovered. So far, 38,589 miRNAs have been identified in different organisms, including 2656 identified in humans, according to the miRBase database [16]. miRNAsMiRNAs are involved in many important processes, such as signal transduction, tissue development, apoptosis [17], proliferation [18], and others [19]. Thus, modulated miRNA expression is associated with various human diseases [20,21]. This has been reported in several studies. For example, B cell chronic anemia is linked to deficiency of the miRNAs miR15 and miR16 [22]. Additionally, in esophageal squamous cell carcinoma, abnormal levels of miRNA expression have been detected, including higher expression levels of miR-25 and miR-223 and lower expression levels of miR-375 [23]. In addition, miR-340 has been proposed as a biomarker for cancer prognosis. The known associations between miRNAs and diseases are documented in various databases, including HMDD [24] and mir2Disease [20,25]. The effects of miRNAs on different disease activities shed light on new treatment perspectives if they can be controlled by small molecules (SMs) [26]. Studies have shown that SMs can activate or repress miRNA transcription; thus, they are being studied as effective treatments for miRNA-related diseases [27,28].

The discovery and production of new small-molecule drugs are always challenging, as they have a high cost and take a long time. Additionally, the efficiency and potential undesired side effects of a discovered drug are not always identified in the early stages. A good alternative is to identify unidentified effects of currently approved drugs for known diseases. This could significantly shorten the path of new drug discovery and reduce costs. If a drug approved for a particular disease is identified as a new potential treatment for another disease, it could directly undergo clinical and toxicity-related studies to obtain faster approval for treating that disease [28]. SMs might act on miRNAs either by directly targeting miRNAs, or indirectly, by targeting related proteins [29]. The identification of the relationships between SMs and miRNAs is expected to support drug repurposing research on miRNA-related diseases [28]. Different SM-miRNA associations have been proven experimentally, revealing the effect of small molecules on miRNA activity. MiR-21 was efficiently inhibited by a diazobenzene derivative, which reduced its transcription. The antibiotic streptomycin was also found to inhibit miR-21 by binding to its precursor [30]. In contrast, the chemotherapeutic agent, 5-fluorouracil (5-FU), was found to increase miR-21 expression levels [31]. Various SMs have been identified as activators or inhibitors of miR-122. The role of miR-122 inhibitors was detected in reducing HCV viral load, whereas miR-122 activators helped decrease the viability of hepatocellular carcinoma cells [32,33]. Other examples include small molecules that have inhibitory effects on miRNAs, such as miR-4644 and miR-27 [34,35].

Traditional drug discovery methods, such as the experimental determination of SM-miRNA associations, consume time and money. Methods used for the experimental detection of SM-miRNA associations include fluorescence detection assays [36,37], luciferase biosensor assays, and the plasmid-reporter-system-based method [35,36].

Computational techniques are vital for the rapid and inexpensive exploration of links between miRNAs and SMs. Recently, computational methods have been used to predict SM–miRNA associations, utilizing different techniques, mostly by calculating similarities between pairs of miRNAs and small molecules [38].

The first attempt to decipher the association between SMs and miRNAs was made by Jiang et al., (2012), who investigated the effects of SMs on the transcription levels of miRNAs in 23 types of human cancers. They created a network, called SMirN, for each cancer type to link small molecules and miRNAs based on their features [39]. Lv et al., (2015) constructed an integrated network by combining SM–miRNA, SM–SM, and miRNA–miRNA associations. The random walk with restart algorithm was applied to the abovementioned networks to assign priorities for miRNAs associated with a given SM [40]. Many studies have followed the similarity scheme used in this study. Another method was applied by Wang et al. in 2016, in which they used the functional similarity of miRNAs and SMs based on the enrichment of differentially expressed genes. They constructed a functional similarity network to predict new miRNA–SM associations. By integrating their results with experimentally proven miRNA–disease associations, they identified 19 potential breast cancer drugs, among which 12 had been suggested in previous reports [41]. Li et al., (2016) presented a framework to predict the effect of anticancer drugs on miRNA regulation, called SMiR-NBI, based on a heterogeneous network scheme that integrates information about miRNAs, small molecules, and genes [42]. Qu et al., (2018) established a HeteSim-based method, called HSSMMA, to infer SM–miRNA associations through a heterogeneous network, using the information available for known SM–miRNA associations and SM/miRNA similarities [43]. Guan et al., (2018) presented a graphlet interaction-based model, GISMMA, for the estimation of SM–miRNA associations by integrating the networks that represent similarities between SMs and miRNAs in addition to known associations. The model assigns scores to new associations using a 28-isomer graphlet interaction model [28].

Many methods followed the same similarity scheme as that of Lv et al., (2015) [40] to predict SM–miRNA associations by integrating biological information from different sources. Lv et al. used four SM similarities: side effects; functional consistency; chemical structure; and indication phenotype similarities, and two miRNA similarities: functional consistency and indication phenotype. The data used to calculate the similarities were retrieved from various websites and databases. Obtaining such data is time- and effort-consuming and cannot be directly possible for newly discovered items. To overcome these problems, we used a different method, partially utilizing deep learning auto-encoders for the automated feature extraction of SMs and miRNAs, without the need to collect data from different resources or domain knowledge.

Deep learning is a widely used technique that has proven to be highly efficient in building prediction models and content creation for different domains, including drug discovery [44,45,46,47]. Auto-encoders are based on a deep-learning-based architecture that is composed of two joint sub-models with the encoder-decoder architecture. The encoder converts inputs into a numerical feature vector, whereas the decoder converts them back to the original form [47]. Both the encoder and decoder are neural networks that are trained together to provide the maximum possible conditional probability for correct outputs. This technique is widely used in statistical machine translation problems, where the inputs are words or characters [48].

A common representation of molecules in the form of strings is the simplified molecular input line entry specification (SMILES) representation [44]. The representation of chemical compounds as SMILES strings enables their modeling through deep learning encoder-decoder models, including recurrent neural network (RNN)-based frameworks [47]. In addition, miRNA sequences can be encoded using such models.

We used the encoder part of an encoder-decoder framework to produce miRNA/small molecule encodings that could replace the traditional representations for calculating similarities. The resulting encoding representations were used to produce similarity matrices using the Euclidean distance. Then, we used these matrices to train a graphlet interaction-based model [28] to predict unknown SM-miRNA associations. To evaluate the efficiency of the new method, we obtained and compared the results of the prediction workflow using similarities produced by our proposed method and those provided by previous work [28]. The results of both methods showed minor differences, with a slight increase or decrease in accuracy for some cases. Our proposed method used auto-encoders to represent the features and predict the associations of small molecules and miRNAs based only on SMILES and miRNA sequences.

## 2. Materials and Methods

### 2.1. Materials

Data were collected for different purposes to construct the similarity matrices and perform SM–miRNA association predictions. We obtained datasets for training the small molecule and miRNA auto-encoders, in addition to the known association dataset that was used to train the prediction model.

#### 2.1.1. Datasets for Training Auto-Encoders

The miRNA auto-encoder was trained on sequences obtained from the RNA Central database (https://rnacentral.org/ (accessed on 10 January 2021)), a free online resource that contains up-to-date lists of non-coding RNA sequences from various organisms. We extracted 35,757 small regulatory human ncRNA sequences, including 3752 miRNAs and 32,005 piRNAs (Piwi-interacting RNA). For small molecules, the auto-encoder was trained on approximately 700,000 SMILES retrieved from the ChEMBL database [49] (https://www.ebi.ac.uk/chembl/ (accessed on 15 January 2021)) for chemical compounds.

#### 2.1.2. Datasets for SM-miRNA Associations

The dataset for the association predictions was obtained from previous works [28,40]. It consists of 831 small molecules (from: SM2miR, DrugBank [50] and PubChem [51]), 541 miRNAs (from: SM2miR [52], HMDD [53], miR2Disease [25], and PhenomiR [25,54]), and 664 known SM–miRNA associations from SM2miR [52].

### 2.2. Methods

SMILES for the 831 small molecules were retrieved from the PubChem database [51]. The miRNA sequences were obtained from the mirBase database [16]. The datasets for miRNAs and small molecules were used to train two auto-encoders using sequences and SMILES, respectively. We produced two encoding versions, 64-d features and 128-d features, for both miRNA sequences and SM SMILES, and the resulting encodings of miRNAs and small molecules were used to produce similarity matrices. Subsequently, the matrices were employed, in combination with known associations, to train the graphlet interaction model and infer unknown associations.

#### 2.2.1. Long Short-Term Memory (LSTM) Sequence Auto-Encoders

**Figure 1:**
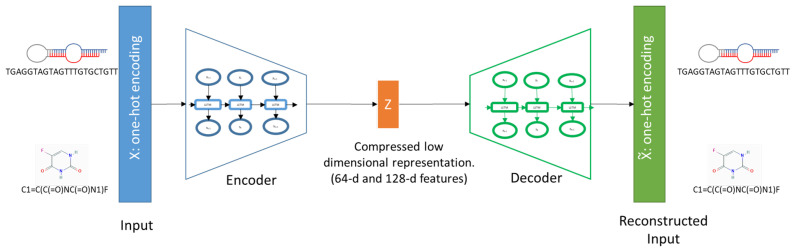
The encoding of miRNAs and small molecules: The input (miRNA sequence/SM SMILES) is represented as one-hot encoding and encoded by the encoder into a compressed low-dimensional representation (64 or 128 features). The decoder part reconstructs the inputs from the encodings to verify the encoding quality.

For sequence encoding, we used sequence-to-sequence learning with an RNN. The encoder represents the first phase of an encoder-decoder framework that relies on a deep neural network and is applied in different domains. The aim of this method was to obtain numerical encodings for the miRNA sequences, or small molecule SMILES (Figure 1).

In general, the encoder-decoder framework works by encoding an input string or sentence with a variable length into a representation of a numerical vector with a fixed length (encoder). The items encoded are processed according to their order over time. The vector is then decoded into a variable-length string or sentence (decoder) [48]. A recurrent neural network encoder-decoder architecture is composed of two RNNs; one works as the encoder, and the other works as the decoder. The method was originally applied to natural language processing and machine translation [55]. RNNs are known to be effective in sequence modeling problems, as they memorize previous sequence items, while trying to predict the following ones [47,56].

We used an LSTM-based encoder-decoder framework that works on the character level of a sequence. The encoder and decoder LSTMs were trained together for each individual dataset. The decoder reproduces the input sequence based on the encoder’s outcome. Hence, the accuracy of the decoder outputs was considered to assess the reliability of the encoder. The ability of the decoder to reproduce the encoded strings indicates its encoding efficiency. Before training the LSTM networks, we built a vocabulary to encode an input sequence into a vector of specific length using one-hot encoding. To train each auto-encoder, we used a different set of possible characters and the maximum length for both SMs and miRNAs. For SMs, a set of 59 possible characters and a maximum length of 50 were considered, whereas for miRNAs, six characters (A, C, G, T, N, and ‘-’) and a maximum length of 30 were used. The outputs of the encoding phase were two encoding versions for each type. The 64-d and 128-d feature vectors were produced. The encoded vectors represent the sequences of miRNAs/SMILES of small molecules for subsequent similarity calculations.

#### 2.2.2. Similarity Calculation

The miRNAs and SMs were represented as numerical vectors based on the encoding process. To estimate the similarity between pairs in each type, we simply used the negated normalized values of the Euclidean distance, per Equation (1). Negation was performed by subtracting the normalized distance value from 1.

(1) $$d_{x,y}=\sqrt{\sum_{d=1}^{D}{\left(x_{d}-y_{d}\right)}^{2}}$$

This equation calculates the distance between two vectors, *x* and *y*, of *D* dimensions. The numbers of dimensions were 64 and 128, respectively, according to the encoding version used. The results of this step are the similarity matrices that were used to replace the ones used by previous researchers. Notably, we obtained the matrices based only on the sequences of miRNAs or SMILES of small molecules, without the need to retrieve or search for other data sources.

#### 2.2.3. Graphlet Interaction

The method used by Guan et al. [28], namely, GISMMA, predicts potential associations between miRNAs and SMs by integrating the calculated similarities for miRNAs/SMs and their sets of known associations. The prediction depends on the graphlet interactions. Graphlet interactions describe the relations between nodes in a specific graphlet, which is a subgraph of a larger network [57]. Graphlets of only four nodes or less were used to establish 28 isomers for calculating the interactions between the nodes. The set of isomers is used to calculate the graphlet interactions between any two nodes, taking into consideration the positions of the nodes involved. The method is described in more detail in previous works [28,57,58]. Similarities were calculated as an integration of different terms representing SM/miRNA properties. Integrated SM similarities included side effects, functional consistency, chemical structure, and indication phenotype similarities, whereas integrated miRNA similarities included functional consistency and indication phenotype.

#### 2.2.4. Predicting Unknown Associations

**Figure 2:**
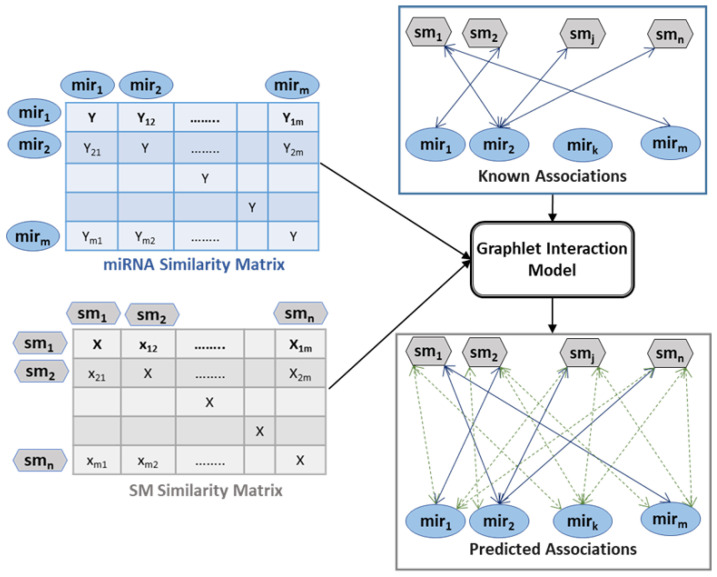
Graphlet Interaction model: similarity matrices and known associations (solid arrows) are inputs; predicted associations (dashed arrows) are outputs.

Similarity matrices were used in combination with known associations to predict new miRNA–SM associations. The graphlet interaction model utilizes input data to assign priority to all possible associations. In a network structure, the model assigns weights to newly established edges between the miRNA and SM nodes, as shown in Figure 2. The highly ranked SM–miRNA associations or edges are predicted to be most likely to exist, whereas a lower rank denotes a lower chance of existing as an actual association.

#### 2.2.5. Evaluation Methodology

To evaluate the prediction efficiency, we partially followed the scheme of a previous work [28]. The accuracy was calculated by determining the ratio of known associations that exists in a specific percentage of top-ranked predictions. The performance was compared based on the priority (rank) given for known associations in each output. The predictions were evaluated and compared for our suggested method and the method used by Guan et al. in [28]. The evaluation was performed using 5-fold cross validation and a set of case studies of both small molecules and miRNAs.

Five-Fold Cross Validation: We used 5-fold cross validation as we randomly divided the known associations into five equal parts. Then, we ran the association prediction five times for each type of investigated similarity. In each of the five runs, we used four parts for training and one part for validation. The accuracy was calculated for each type as the average accuracy resulting from the five runs. Accuracies were determined for different percentage points of the top predicted associations.

Validations of Case Studies: For further evaluation and comparison of the proposed method’s performance in specific cases, we carried out a variety of case study validations. As a general flow in the case study validations, we extracted the known associations of a given item, a small molecule, or an miRNA, and removed them from the known associations. The extracted associations were kept away to validate the predictions by identifying how highly they were ranked in the predicted associations. For each case study, we compared the predictions based on the Guan et al. similarity method to the encodings-based similarity of the 64-d and 128-d feature vectors. The comparison was performed by calculating the percentages of known associations found at different top percentage points in the ranked predictions. We considered six SMs as case studies based on the frequency of known associations. Information about the six compounds were retrieved from the PubChem database [59] (https://pubchem.ncbi.nlm.nih.gov/ (accessed on 15 February 2021)).

- 139: Enoxacin (CID: 3229): An antibacterial drug that inhibits DNA synthesis. It is used to treat gonorrhea and urinary tract infections.
- 162: 5-Fluorouracil (CID: 3385): An antineoplastic agent that inhibits DNA synthesis and is also used for treating solid tumors that occur in different body parts, such as the breast, colon, and liver.
- 351: Vorinostat (CID:5311): An antineoplastic agent and a deacetylase inhibitor, which is used for treating cutaneous T cell lymphoma.
- 405: Estradiol (CID: 5757): A synthetic form of the steroid sex hormone, estradiol, which maintains fertility and female characteristics. Synthetic estradiol can be used as a hormone replacement therapy.
- 607: Gemcitabine (CID:60750): An antineoplastic agent used for treating advanced lung, breast, and pancreatic cancers.
- 736: Diethylstilbestrol (CID:448537): Used in the treatment of prostate and breast cancers.

As an miRNA case study, we applied the same workflow to check the predicted associations for miR-21. The number of associations for one miRNA among the known associations was notably much lower than that of small-molecule associations. We selected miR-21 as a case study because miR-21 had the highest number of small-molecule associations (10) in the dataset of known associations.

## 3. Results

### 3.1. Evaluation

Applying the evaluation methodology described in the previous section, we assessed the accuracy of the predictions of our suggested method. The performance of our method was compared to the method applied by Guan et al. [28]. Our method achieved better or the same accuracy predictions in most of the cases and minor or less accuracy in very few cases. We describe and compare the results of different similarity calculation methods in this section.

#### 3.1.1. 5-Fold Cross Validation

The results for the 5-Fold cross validation evaluation are shown in Table 1; the first column shows the percentage points considered for the evaluation. The ratio of correctly predicted (known) associations that were found within the corresponding top percentage points are shown in the second, third, and fourth columns, for the Guan et al. [28], 64 d vector, and 128 d vector similarities, respectively.

**Figure 3:**
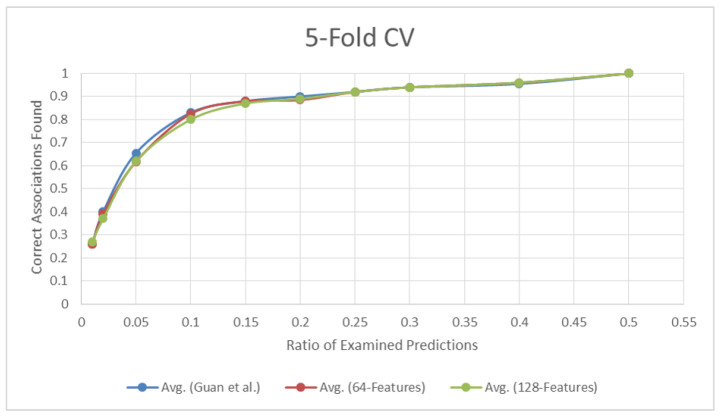
Percentages of Correct Predictions for 5-Fold Cross Validation for Guan et al. 64-d Features, and 128-d Features.

Figure 3 plots the accuracy values for the 5-fold cross validation at the selected percentage points when applied to different similarities. The difference in accuracy appears slightly in the percentage points between 0.01 and 0.15, although it is not apparent for the remaining points.

#### 3.1.2. Case Study Results

The average accuracy values for the six small-molecule case studies are shown for the selected percentage points in Table 2. At most points, the accuracy values of the 64-d feature similarity were the highest. The differences in accuracy were clearer for percentage points from 0.02 to 0.2. In the top 15% of predicted associations, the 64-d feature similarity predictions had 10% more confirmed associations than the Guan et al. similarity predictions.

**Figure 4:**
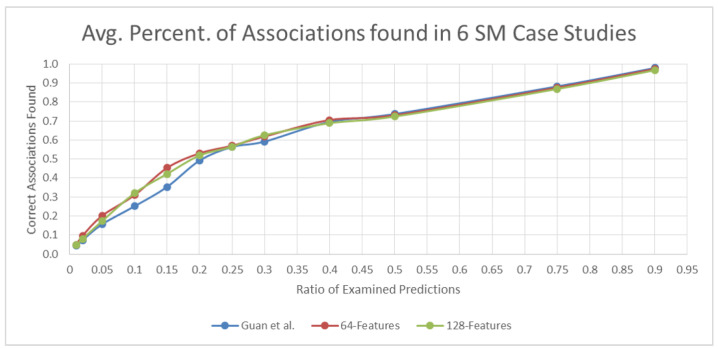
Avg. prediction curves for 6 SM case studies at different points (Guan et al., 64-d feature, and 128-d feature methods).

Figure 4 plots the curves for the performance of each similarity method at different percentage points. Additional results obtained for each case study are available in the Supplementary materials (Table S1).

**Figure 5:**
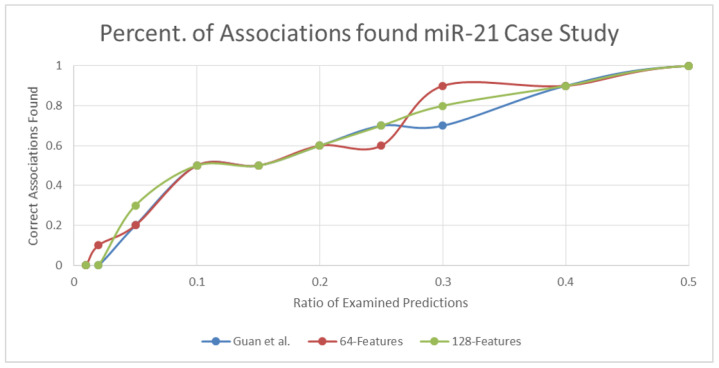
Avg. prediction curves for the miR-21 case study at different points (Guan et al., 64-d, and 128-d feature methods).

In the miR-21 case study, predictions of encoding-based similarity methods performed similarly to or better than the similarity method used by Guan et al. At only one percentage point, 0.25, the 64-d feature prediction had 10% fewer confirmed associations, indicating that only one association was absent within this portion. Figure 5 plots the performance of the different similarity methods for the miR-21 case study.

Four molecules that were examined in the small-molecule case studies were present in the known associations for miR21 (enoxacin, 5-fluorouracil, vorinostat, and gemcitabine). By locating these SMs in the predictions of miR-21 associations, they were found to be within the top 9% of associations.

## 4. Discussion

Most of the studies that have tried to computationally associate SMs and miRNAs rely on calculating similarities between features collected from different sources. Our objective was to replace this time- and effort-consuming process with an automated method, while maintaining at least the same efficiency in association predictions. Our overall methodology was designed to allow a direct comparison of our results with those obtained by Guan et al. To enable this comparison, we evaluated the results of both methods based on the fact that finding more known associations at smaller percentage points in the top-ranked predictions indicates better predictability. From this perspective, we analyzed and compared the results in the tables. We evaluated and compared the overall performance using 5-fold cross validation in addition to case-based evaluations using six small molecules and one miRNA. The 5-fold cross validation values in Table 1 show slight differences between the similarity types for most of the percentage points. The accuracies in the table show that, at most of the percentage points, the 64-d feature vector performed better (points: 0.02, 0.10, 0.15) or equally well (points: 0.05, 0.20, 0.25, 0.30, 0.40, 0.50), when compared to the 128-d feature vector. When we compare the 64-d feature vector similarity method to the Guan et al. similarity method, we can see the highest difference, 0.04, at percentage point 0.05. Other than at this point, the accuracies were almost equal. In the case study validations, both encoding-based similarities performed better than the Guan et al. similarity. The average values over the six small molecules case studies showed better predictions for the 64-d feature similarity. Not only did the suggested encoding-based similarity method maintain accuracy at reasonable levels, but it also produced better predictions for the case studies in the top-ranked associations. Better predictions in the top-most items can give more confidence when selecting new unconfirmed associations for specific cases. The same also applies for the single miRNA case study, miR21. The encoding-based similarity methods performed similarly to or better than the similarity method used by Guan et al. at almost all percentage points. Additionally, we found four of the case study molecules within the confirmed associations of miR21. When locating these four SMs in the 64-d feature similarity predictions, they appeared within the top 9% of predictions. The results of the different validation methods show efficient ranking and high predictability of the SM–miRNA associations based on 64-d feature similarity method.

## 5. Conclusions

In this study, we proposed a deep-learning-based method for the faster detection of associations between miRNAs and SMs, relying only on compound SMILES and miRNA sequences. The suggested method was applied in conjunction with a previously used scheme on one of the widely used datasets but with an alternative similarity calculation approach. We calculated the similarity between miRNAs/small molecules after representing them as numerical feature vectors produced by deep learning auto-encoders. Then, these similarities were used with the previously known SM–miRNA associations to train a graphlet-based network model designed to infer new potential associations. The numerical encoded feature vectors replaced the time-consuming step of collecting biological information from multiple sources. The evaluations showed a minor difference in the prediction ability between the new and previous similarity schemes. The best performance was obtained with the 64-d feature representation, which achieved better predictions in most points for cross validation and case study evaluations. The results proved the effectiveness of our proposed method and its ability to replace the time- and effort-consuming traditional methods for similarity calculation.

## Figures and Tables

**Table 1:** Average ratio of known associations found during 5-fold cross validation at different percentage points for Guan et al., 64-d feature, and 128-d feature similarities.

Percent	Guan et al.	64-d Features	128-d Features
0.01	0.26	0.26	0.27
0.02	0.40	0.39	0.37
0.05	0.66	0.62	0.62
0.10	0.83	0.83	0.80
0.15	0.88	0.88	0.87
0.20	0.90	0.89	0.89
0.25	0.92	0.92	0.92
0.30	0.94	0.94	0.94
0.40	0.96	0.96	0.96
0.50	1.00	1.00	1.00

**Table 2:** Average ratio of known associations found during 6 SM case studies at different percentage points for Guan et al. 64-d features, and 128-d features similarities.

Percent	Guan et al.	64-d Features	128-d Features
0.01	0.04	0.05	0.05
0.02	0.07	0.10	0.08
0.05	0.16	0.20	0.17
0.10	0.25	0.31	0.32
0.15	0.35	0.45	0.42
0.20	0.49	0.53	0.52
0.25	0.56	0.57	0.57
0.30	0.59	0.62	0.63
0.40	0.70	0.71	0.69
0.50	0.74	0.73	0.72

## Data Availability

All the source code and data are available as a Github repository at https://github.com/ibrahimzb/SMMIR-CFRAutoEnc, (accessed on 5 November 2021).

## Supplementary Materials

The following are available online at https://www.mdpi.com/article/10.3390/pharmaceutics14010003/s1, Table S1: Accuracy values for 6 small molecule, and 1 miRNA case studies.
